# Symptoms, work situation and work functioning 10 years after rehabilitation of stress-induced exhaustion disorder

**DOI:** 10.1186/s12888-024-05975-x

**Published:** 2024-07-23

**Authors:** Therese Eskilsson, David Olsson, Anna-Maria Ekbäck, Lisbeth Slunga Järvholm

**Affiliations:** 1 Department of Community Medicine and Rehabilitation, Physiotherapy, Umeå University, Umeå, Sweden; 2 Department of Public Health and Clinical Medicine, Sustainable Health, Umeå University, Umeå, Sweden

**Keywords:** Stress-induced exhaustion disorder, Burnout, Mental health recovery, Return to work, Rehabilitation, Follow-up studies

## Abstract

**Background:**

Stress-induced exhaustion disorder (SED) is the most common reason for long-term sick leave in Sweden and the recovery process may be long and troublesome. This study explores the symptoms of burnout, depression and anxiety among patients with SED 10 years after termination of a multimodal rehabilitation program. Another aim of the study was to investigate work situation, work functioning, and any remaining exhaustion and sleeping disorders among those who were gainfully employed at the 10-year follow-up.

**Methods:**

This longitudinal study included 107 patients (91 women and 16 men), who had been diagnosed with SED 10 years prior to the study. After establishing the diagnosis they all underwent and completed an multimodal rehabilitation program. Data on symptoms of burnout, anxiety and depression were collected before and after the multimodal rehabilitation program, and at follow-ups after additional 1 year and an additional 10 years. At the 10-year follow-up, work situation, work functioning, and symptoms of exhaustion and sleep disorders were assessed in those who were gainfully employed (89 patients).

**Results:**

Symptoms of burnout, anxiety, and depression remained stable from the 1- to the 10-year follow-up after completed rehabilitation. Among participants who were gainfully employed, 73% had changed workplaces, and 31.5% had reduced their working hours. Common reasons for these changes were lack of energy or because they had chosen to prioritise their lives differently. Work functioning was rated as moderate, one third self-reported SED to some extent, and one fifth reported moderate-to-severe insomnia.

**Conclusion:**

A relatively large proportion of former patients with SED have residual health problems 10 years after rehabilitation and some have not been able to return to full-time work. Preventive and early rehabilitative interventions with adjustments and measures at the organisational level are probably needed to achieve a more sustainable working life.

## Background

During the last few decades, sick leave for mental ill-health has increased in many countries, and the sick absence tends to be long-term in many cases [1]. In Sweden, psychiatric diagnoses have become the most common reason for sick leave in both women and men [2]. The psychiatric diagnosis that has increased the most is stress-induced exhaustion disorder (SED), classified as an illness in the Swedish version of the ICD-10 (diagnostic code F43.8A). SED has been implemented in clinical practice and seems to be a valid clinical equivalent of burnout [3, 4].

Persons with SED describe severe exhaustion, lack of endurance, and a prolonged recovery time after mental effort [3]. Core symptoms are sleep disturbances and cognitive impairments [4], and previous studies have shown that persons with clinical burnout perform worse than healthy controls in multiple cognitive areas [5]. A variety of psychiatric and somatic symptoms and decreased quality of life have also been presented [6].

Rehabilitation of SED often includes different unimodal or multimodal interventions. Some studies have reported reduced symptoms and decreased sick leave after rehabilitation compared to controls, but the benefits were not maintained at follow-ups [6]. Only two randomised controlled trials have demonstrated long-lasting effects, with reduced symptoms of burnout after rehabilitation with cognitive behavioural therapy [7], and a small improvement in global cognitive functioning after adding cognitive training to a multimodal rehabilitation (MMR) program [8]. However, there is still no consensus regarding optimal rehabilitation of SED. In a recent study, we showed that healthcare consumption remains at a high level one-year after participating in a MMR program [9].

Quantitative and emotional demands at work (e.g. workload, time pressure, overtime work and lack of resources or competence for work tasks and assignments) are among the most common self-reported stressors in SED [10]. From a preventive perspective, the workplace is important since psychosocial risk factors at work (high demands, low job control, high work load, low reward and job insecurity) [11] and presenteeism with reduced work performance [12] increase the risk of developing exhaustion. Working conditions and coping strategies at work also play a significant role in sick leave level for patients on long-term sick leave due to SED. This was reported by Norlund et al. [13] where those who reported low control at work and who used covert coping mechanisms (e.g. to what extent a person uses an avoidant behaviour when experiencing work conflicts) towards supervisors and/or workmates had a higher risk of not reducing their sick leave after rehabilitation.

Over time, most persons with SED (87%) return to work or do not receive sickness benefit, as shown in a previous clinical study who included patients with SED seven years after participating in an MMR program [14]. Nevertheless, a large proportion in the study reported residual symptoms, and one third were judged to have SED [14]. In the same study population, it was reported that 63% had enacted some change of their work situation, and gender differences were found. Men changed work tasks to a greater extent and women more often reduced their working hours [15]. In a previous 3-year follow-up after SED rehabilitation, we also found that a large proportion of persons in part-time and full-time work reported mental (63%) and physical (61%) fatigue after work [7]. To conclude many persons that have previously been treated for SED still seem to have residual symptoms and need to make adjustments to their work situation. We consider it important to investigate the course of symptoms, work situation and work functioning over an even longer perspective for this group, because these conditions have a major impact on individuals, employers, and societal costs.

### Aim

The aim of this study was to explore long-term course of symptoms of burnout, depression, and anxiety among patients with SED 10 years after termination of an MMR program. Another aim was to investigate work situation, work functioning and any remaining exhaustion and sleeping disorders among those who were gainfully employed at the 10-year follow-up.

## Methods

### Study design and participants

**Fig. 1:**
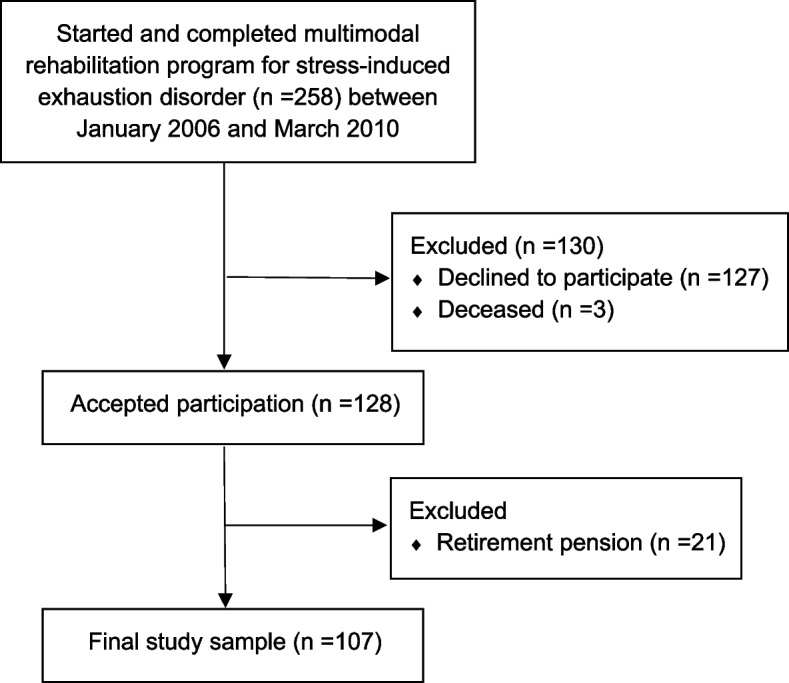
Flowchart of the patients with stress-induced exhaustion disorder that were referred to the Stress Rehabilitation Clinic and followed until 10 years after a completed multimodal rehabilitation program

A longitudinal study was conducted at the Stress Rehabilitation Clinic, University Hospital of Umeå. All participants were former patients diagnosed with SED who participated in an MMR program at the clinic, and who were not involved in other intervention studies. Inclusion criteria for participating in this study was to have started and ended the MMR program including the 1-year follow-up, during the period spanning January 2006 until the end of March 2010. In total, 258 patients fulfilled the inclusion criteria, and of these 128 patients (50%) agreed to participate. Since we wanted to investigate those who were of working age, we excluded those who reported a retirement pension (*n* = 21). The final study population consisted of 107 patients (91 women and 16 men), with a mean follow-up time of 10.4 years (SD 1.07) and a range follow-up time of 9–13 years after the MMR program was completed. A flowchart of patients in the study is presented in Fig. 1.

The MMR program consisted of group-based cognitive behavioural therapy (CBT), individual physical activity prescription, and vocational measures; including a rehabilitation meeting where the physician, the patient, the employer, and an official from the Swedish Social Insurance Agency participated. Each CBT group consisted of eight patients and the aim of CBT was to support behavioural change. Each group focused on specific themes during the three-hour sessions, such as psychoeducation regarding stress, emotions, and recuperation behaviours. During 2006 until mid-2007, the CBT group met weekly in 30 sessions, after which the CBT program was shortened to 24 sessions. There were also short follow-up meetings at 3, 6, and 12 months after the end of the MMR program. The MMR program has been described in more detail earlier [7].

Ethical approval for this study was confirmed by the Regional Ethical Review Board in Umeå, Sweden (Approval Nr. 218/387–31) and was conducted in accordance with ethical principles of the Declaration of Helsinki. All participants received written information about the study and provided written informed consent before entering the study.

### Procedure

During the MMR program patients answered questionnaires including information about symptoms of burnout, depression, and anxiety before starting the MMR program (preMMR), after completing the MMR program (postMMR), and at one-year follow-up (1YFU). To evaluate long-term prognosis at the 10-year follow-up (10YFU) the same questions were included in the questionnaire. At 10YFU, all participants also answered questions about sex, age, educational level, employment status, and levels of sick leave. Variables of work situation, work functioning, self-reported SED, and insomnia were also assessed in those who were working.

### Measurements

#### Burnout, anxiety and depression

*The Shirom-Melamed Burnout Questionnaire* [16] was used to measure level of burnout/exhaustion. It consists of 22 items, each rated on a 7-point Likert scale (1 = “almost never”; 7 = “almost always”). An overall index was calculated as the mean of all items, where a higher score indicates a higher level of burnout. A total mean score of ≥ 4.4 has been suggested as a cut-off for clinical burnout [17].

*The Hospital Anxiety and Depression Scale* was used to measure symptoms of anxiety and depression [18]. The questionnaire consists of 14 items, rated on a 4-point Likert scale (0–3), with seven items addressing symptoms of anxiety and depression, respectively. A total score on each scale (range: 0–21 points) was calculated, with a higher score indicating more symptoms.

#### Sick leave

The patients were asked to report if they were on sick leave and on what level (0%, 25%, 50%, 75%, 100%). They could also indicate the type of financial compensation they had during their sick leave (sickness benefit or sickness compensation from Social Insurance Agency or sickness compensation from employer).

#### Variables for those at work

*Current work situation and work tasks* compared to 10 years ago was assessed by asking the participants to indicate one of six options; “Do you work 1; at the same- or 2; different workplace with the same tasks as before?”, “Do you work 3; at the same- or 4; different workplace with new tasks?”, and”Do you work 5; at the same- or 6; different workplace with adapted tasks?” A question was also asked about reduced working hours/week and, if so, for what reason.

*The Work Role Functioning Questionnaire 2.0* was used to assess perceived difficulties in meeting work demands due to physical health or emotional problems. This questionnaire consists of 27 items, and examples of items are; I found it difficult “to work fast enough”, “to concentrate on work” or “to prioritise in my work”. Difficulties in meeting work demands were estimated during the previous four weeks on a 5-point scale (0 = difficult all the time; 1 = difficult most of the time; 2 = difficult half of the time; 3 = difficult some of the time; 4 = difficult none of the time). There is also a response option “does not apply to my job” which was classified as a missing value and were excluded from the analysis. A total score on each of the subscales;—work scheduling and output demands (WSOD), physical demands (PD), mental and social demands (MSD), and flexibility demands (FD) —was calculated as the average of items and multiplied by 25 to obtain a score between 0 and 100%, where higher scores indicate better work functioning [19]. The score could also be classified as high (> 90), moderate (75–89), and low (< 75) work functioning [20]. If 20% or more items were missing in each subscale, it was classified as missing [19].

*Self-rated exhaustion disorder (s-ED)* was used to classify self-rated SED at 10YFU. The s-ED is based on diagnostic criteria for SED and consists of four items; 1) “do you currently feel, and have felt for more than 2 weeks, physically and/or mentally exhausted?”, 2)“do you consider this exhaustion to be caused by long-term stress exposure (that you have been exposed to great strain or experienced pressure for 6 months or more)?” 3) “during the last 2 weeks, have you experienced a) concentration or memory problems, b) markedly reduced capacity to tolerate demands or to work under time pressure, c) emotional instability or irritability, d) sleeping problems, e) physical weakness or being more easily fatigued, f) physical symptoms, and 4) “have the complaints (items 1–3) markedly decreased your well-being and/or your functional capacity (work ability, family life, leisure activities or in other important ways)?” Participants confirming the claims in the first, second and fourth item, as well as acknowledging four of the six conditions in the third item were classified as having SED. The fourth item also discriminates between light/moderate (yes, somewhat) and pronounced (yes, to a great extend) SED [21].

*Insomnia Severity Index* was used to assess the severity and impact of insomnia. The insomnia severity index consists of seven items, rated on a 5-point Likert scale (0 = “no problem”; 4 = “very severe problem”). A total score was calculated (range 0–28 points) and was interpreted as: absence of insomnia (0–7); sub-threshold insomnia (8–14); moderate insomnia (15–21), and severe insomnia (22–28) [22].

### Statistics

Analyses of characteristics data and the differences in responders and non-responders were made by using t-tests for continuous data and Pearson’s χ2 tests for categorical variables. Repeated measures ANOVA were used to test for any overall differences in the outcomes. Post hoc Bonferroni corrected pairwise t-tests were used to compare changes in symptoms during rehabilitation and follow-up. A p-value less than 0.05 was considered statistically significant. All analyses were performed using R statistical software (R Core Team, 2022) [23].

## Results

In this 10-year follow-up (10YFU), a total of 107 patients (91 women and 16 men) answered the questionnaires. There were no significant differences between responders and non-responders regarding sex, sick leave, or level of burnout before starting the MMR program. However, a significant difference was seen in age before starting the MMR program between responders (mean age of 52.9; SD 7.2) and non-responders (mean age of 45.4; SD 9.0), (*p* < 0.001). Responders also showed a significantly larger recovery in rates of burnout (SMBQ) (–1.4; SD 1.2) during the MMR program compared to non-responders (–1.07; SD 1.07), (*p* = 0.026).

### Demographic characteristics and sick leave data

Demographic characteristics of the patient group and levels of sick leave at 10YFU are presented in Table 1. Most of the patients were employed (76%) and some were self-employed (9%). The majority were not on sick leave (78%); 13% were on part-time sick leave and 9% reported full-time sick leave. In total, 24 patients reported sick leave either part-time or full-time. Of these, 13 had stated which diagnosis was the cause of the sick leave; 7 patients reported SED and the remaining diagnoses reported were neck and back pain (*n* = 1), breast cancer (*n* = 1), bipolar disorder and heart failure (*n* = 1), nerve pain and cancer (*n* = 1), allergy (*n* = 1), and trigeminal neuralgia (*n* = 1).

**Table 1:** Demographic characteristics and levels of sick leave at 10-year follow-up after participating in a multimodal rehabilitation program for stress-induced exhaustion disorder

Variable	(*n* = 107)
Sex (women/men)	91/16
Age, mean (SD)	52.89 (7.24)
Education, n (%)	
University	63 (59)
Employment status, n (%)	
Employed	81 (76)
Self employed	10 (9)
Unemployed	16 (15)
Sick leave, n (%)	
100%	10 (9)
75%	2 (2)
50%	5 (4.5) ^a)^
25%	7 (6.5) ^a)^
0%	83 (78)

^a)^Compensation from employer, *n* = 1

### Symptoms of burnout, anxiety and depression

**Fig. 2:**
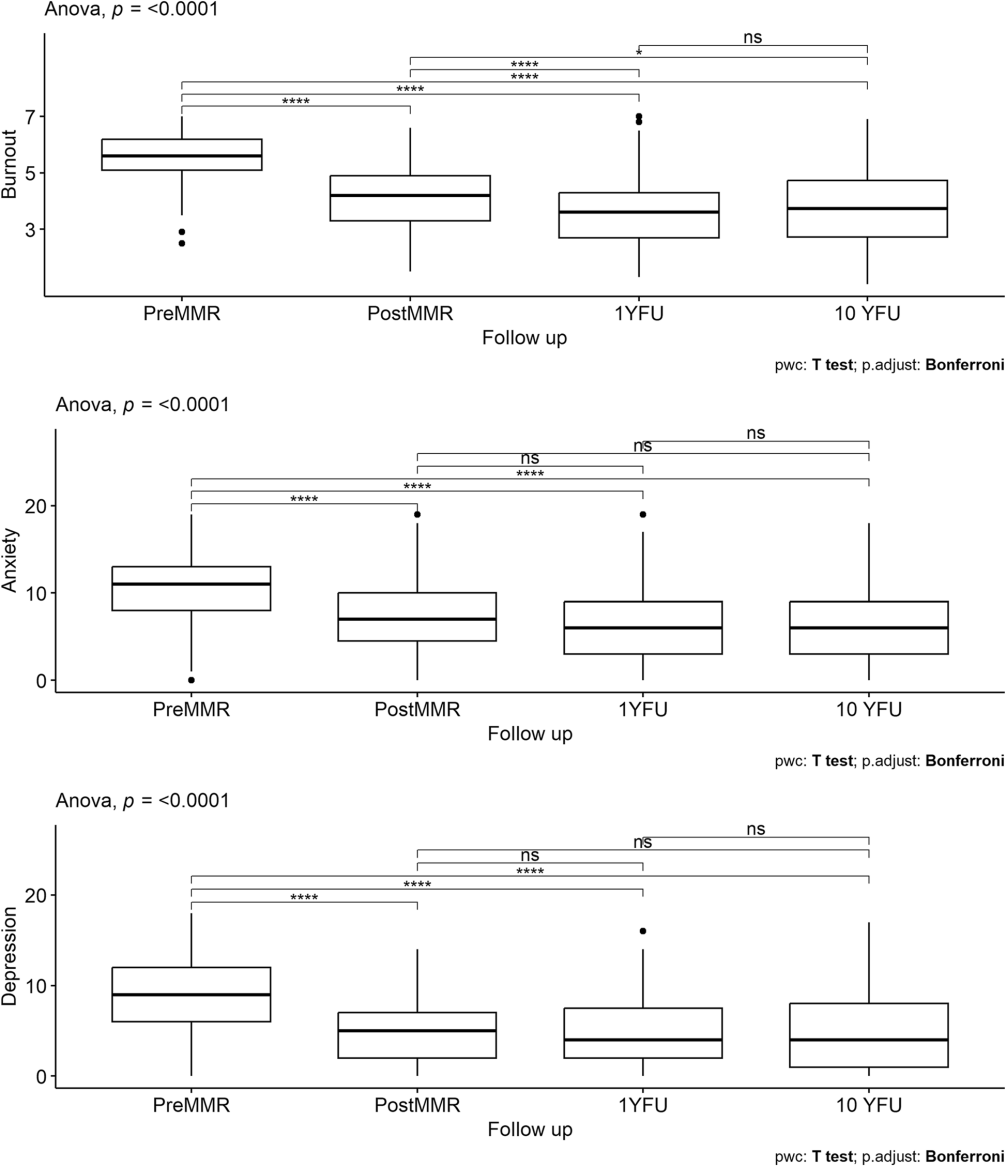
Boxplots for burnout, anxiety, and depression. p-value for overall differences in the top right of each panel. Bonferroni corrected significance levels for pairwise t-test are ns = non-significant, * < 0.05, ** < 0.01, *** < 0.001, **** < 0.0001

Reported levels of symptoms showed a statistically significant improvement during the MMR program, concerning burnout, anxiety, and depression (*p for overall difference* < 0.01 for all outcomes). The scores for all outcomes improved from preMMR to postMMR (Bonferroni corrected p-values < 0.01 in all pairwise comparisons), and the burnout score improved also to the 1YFU and 10YFU (*p*-value < 0.01 when comparing postMMR with 1YFU, and 10YFU respectively). No further statistically significant improvements were seen in the other variables from the postMMR, to the 1YFU or the 10YFU. Development of levels of burnout, anxiety, and depression throughout the MMR program and follow-ups are shown in Table 2 and Fig. 2.

**Table 2:** Mean scores and standard deviations of burnout, anxiety, and depression during the multimodal rehabilitation program and long-term follow-up at 10-year (*n* = 107)

Variable	Mean (SD)			
	PreMMR	PostMMR	1YFU	10 YFU
**Burnout**	5.54 (0.90)	4.13 (1.13)	3.64 (1.22)	3.76 (1.36)
**Anxiety**	10.71 (4.18)	7.35 (4.11)	6.51 (4.16)	6.39 (4.51)
**Depression**	8.79 (3.89)	5.33 (3.62)	4.64 (3.72)	4.67 (3.82)

Burnout was measured by SMBQ (range 1–7), anxiety and depression by HADS (range 0–21)

*MMR* multimodal rehabilitation, *1YFU* 1-year follow-up, *10YFU* 10-year follow-up

### Work situation and work functioning for those at work

A total of 89 patients (76 women, 13 men) reported that they were working (*n* = 86) or studying (*n* = 3), part-time or full-time. Those who worked part-time could also be job-seekers, on sick leave, or had not specified another activity. A large proportion (73%) had changed workplaces after the MMR program, either to work in similar tasks (*n* = 23), completely new tasks (*n* = 34), or adapted tasks (*n* = 8). Of those who remained in the same workplace, only a few had new (*n* = 5) or adapted work tasks (*n* = 2). About 30% reported that they had reduced their working hours. The reason for the reduced working hours was stated to be that their mental and physical energy was not sufficient for full-time work or that they prioritised their lives differently today than they had a decade ago. Work functioning was classified as moderate in all subscales, with the lowest reported work function in work scheduling and output demands. In Table 3 results are reported for those who are working.

**Table 3:** Work situation, work functioning and self-reported symptoms at 10-year follow-up in those who are working

Variable	(*n* = 89)
Work situation, n (%)	
New workplace	65 (73)
New work tasks	39 (44)
Adapted work tasks	10 (11)
Reduced working hours ^n=88^	28 (31.5)
Work functioning, mean (SD)	
Work scheduling and output demands ^n=79^	77.69 (26.55)
Physical demands ^n=63^	82.54 (26.60)
Mental and social demands ^n=86^	78.65 (20.29)
Flexibility demands ^n=83^	80.56 (21.19)
Self-rated exhaustion disorder, n (%)	
None	61 (68.5)
Light/moderate	13 (14.5)
Pronounced	15 (17)
Burnout ≥ 4.4, n (%)	25 (28)
Insomnia, n (%)	
Absence of insomnia	40 (45)
Sub-threshold insomnia	29 (32.5)
Moderate insomnia	15 (17)
Severe insomnia	5 (5.5)
Total score, mean (SD)	8.94 (6.38)

Work functioning was assessed by the Work Role Functioning Questionnaire, self-rated exhaustion disorder by s-ED, burnout with Shirom-Melamed Burnout Questionnaire, and Insomnia with Insomnia Severity Index

### Self-reported symptoms for those at work

About 30% self-reported SED (s-ED) to some extent and scored above the clinical cut-off level for burnout/exhaustion (SMBQ). Moderate-to-severe insomnia occurred in about one-fifth of those at work (Table 3).

## Discussion

This long-term follow-up until 10 years after completed rehabilitation of SED shows that symptoms of burnout, anxiety, and depression level out and remain stable from the 1-year follow-up, but on a slightly higher burnout level than in the general population of the same age [24]. Among those who were working, a large proportion had changed workplaces, work functioning was rated as moderate, one third self-reported SED to some extent, and one fifth reported moderate-to-severe insomnia.

It is gratifying that the symptoms are still stable after such a long time, and the continued course is similar to our previous 3-year follow-up [7], but the results also show residual problems with a fifth of the persons still on sick leave to some degree. However, the reason for sick leave was not exclusively SED, as described in a previous long-term follow-up [14]. Some of our participants indicated SED as a reason for sick leave, but other diseases that can be considered common in a normal population were also reported.

As we lack a control group, we cannot comment on the prolonged effect of rehabilitation, about which there is still a lack of consensus [6]. However, our results are interesting, as they provide an increased understanding of continued health and the work situation for persons who had previously been treated for SED. Surprisingly, many participants (73%) had changed workplaces, in comparison to the general Swedish population, where 25.5% changed workplaces during a 5-year period [25]. The proportion in our study who changed workplace was also higher than in a previous cross-sectional study which investigated work situations among former patients seven years after rehabilitation for SED [15]. In that study, it was reported that 47% changed workplaces, and 42% changed work tasks because of their illness [15], which is similar to our results. Work-related stress such as conflicts, reorganisations, deficient leadership, and discontent with the work situation have previously been reported as reasons for patients with SED to implement changes at work [15]. Unfortunately, we do not have information about the reasons for changing workplace or work tasks in our study. However, factors that contribute to the development of SED may not be the same as those that contribute to maintenance of SED, where contextual factors and behaviour may play a role [26]. This point may be important, because despite a new workplace, and new or adapted work tasks, 31.5% of the participants in our study still self-reported SED and sleep disturbances, which likely contributes to a continued chronic lack of recovery [4]. The decreased energy and prolonged recovery time that characterises SED appears to persist, and to deal with this, some participants may have chosen to reduce their working hours. However, there were also some persons who reduced working hours on the basis that they now prioritised their lives differently; perhaps because of the previous CBT rehabilitation.

Persons with SED have also described in qualitative studies that recovering from SED is hard work [27], and even after 10 years they still struggled with dysfunctional attitudes and strategies, stress sensitivity, and cognitive impairments [28]. This condition, of course, not only affects their private situation but also their working lives. A previous study on employees with common mental disorders showed high presenteeism-related productivity loss before, during, and after sick leave [29], which probably also applies to persons with prolonged SED.

Participants in our study reported limited work functioning and difficulties meeting work demands to a higher degree than the general working population [19]. The work demands that were most difficult to manage were work scheduling and output demands, and mental and social demands, which were nearly classified as low work functioning. This is also consistent with previous studies where persons with SED reported long-term cognitive impairments [28], and impaired cognitive performance [30, 31]. They also need to increase their mental effort during complex cognitive tasks, which subsequently results in increased fatigue [32]. With this as a background, it is of great importance that persons with residual symptoms of SED receive support in making adjustments, particularly in cognitively demanding work tasks. In our study, only 10 persons had adapted work tasks and probably many more would have needed this action.

In Sweden, employers are responsible for the work environment and should prevent risks of ill health and make adjustments when necessary [33]. Unfortunately, employers probably often lack knowledge and strategies for how to act to prevent and rehabilitate employees with mental health problems [34]. In Sweden, a workplace-oriented intervention for persons on sick leave due to SED has been developed [35]. The intervention is a 3-step interview model, where a health care provider coordinates a dialogue between the employee and the manager in order to find concrete actions for sustainable work ability or for return to work. For participants on sick leave due to SED, the intervention has shown positive effects on return to work [35], fewer sick leave days compared to a control group [36], and has proven cost-effective from a welfare perspective [37]. Managers' experience of the intervention is that they are strengthened in their ability to act in the return to work process [38]. A similar workplace intervention in occupational health service among employees with common mental disorders or stress-related symptoms has also been shown to significantly reduce sickness absence and supported faster partial return to work compared with care as usual [39]. To actively implement adjustments at work should be of central importance to employers, because remaining symptoms might cause production loss at work [40]. Overall, it seems that SED is frequently work-related and that workplace interventions that actively involve both employees and managers in concrete adjustments to work tasks can facilitate a sustainable return to work.

### Limitations and strengths

This study has several limitations that should be mentioned. At first, lack of a control group is a significant limitation of this study, thus ruling out possible causal inferences between treatment and symptom improvements. However, clinical studies such as this long-term follow-up can be of great value for further evaluation of treatment and, above all, new knowledge about how the patients' work situation and work function are 10 years after rehabilitation.

Furthermore, the response rate was only 50%, and there were significant differences between responders and non-responders in age and recovery rate. This may introduce some selection biases that could limit the generalisability to persons in general with SED. Responders in this study were older, but they also showed a larger recovery rate in rates of burnout during the MMR program. Our results may therefore reflect a more positive picture of the participants' work situation, since the responders probably constituted a "healthier" population. Furthermore, older people do not change jobs to the same extent [41]. However, our results are in line with previous long-term follow-ups, where approximately the same proportion were judged to still meet the criteria for SED [14]. The study was conducted at a Stress Rehabilitation Clinic, which is specialist care, and the results are likely not fully generalisable to patients with SED in primary care who may have less severe SED. Another limitation is the use of self-reported measures. Most of the measurements used are based on valid instruments, but we know that e.g., register-based data on sick leave would have been more secure and preferable.

On the other hand, our results complement important information about long-term follow-up of people who have previously been treated for SED, and the situation for those who are still in work. The results should be important to consider in work-related rehabilitation in order to prevent continued problems with SED, and to promote sustainable return to work.

## Conclusions

In this study, we found that symptoms of burnout, anxiety, and depression remain stable at 10 years after completion of an MMR program for SED. A remarkable proportion of the participants (73%) had changed workplaces, but despite this, their work situation seems to be burdened in different ways. Work functioning was rated as moderate, a third had reduced their working hours, and a considerable proportion still self-reported SED to some extent (31.5%), in addition to moderate-to-severe insomnia (22.5%). Despite this, quite few persons had adapted work tasks, even though it is the employer's duty to prevent risks of ill-health and make adjustments if necessary. Further studies need to more closely examine how interventions can focus on the work situation, and measures should be taken at the organisational level to make adjustments that enable full recovery from SED and promote continued health.

## Data Availability

The datasets analysed during the current study are available from the corresponding author on reasonable request.

## Abbreviations

SED: Stress-induced exhaustion disorder
MMR: Multimodal rehabilitation
CBT: Cognitive behavioral therapy
YFU: Year follow-up
s-ED: Self-rated exhaustion disorder
